# Various surgical techniques result in different outcomes for full extensor mechanism ruptures following total knee replacement: A systematic review by the European Knee Associates (ESSKA‐EKA)

**DOI:** 10.1002/ksa.70015

**Published:** 2025-08-29

**Authors:** Bruno Violante, Riccardo Compagnoni, Artur Kroell, Michael Engl, Octav Russu, George Mihai Avram, Sarper Gursu, Elvire Servien, Francesco Puglia, Pietro Simone Randelli, Reha Tandogan, Michael T. Hirschmann, European Knee Associates (EKA)

**Affiliations:** ^1^ Director Orthopaedic Department Fatebenefratelli Isola Tiberina – Gemelli Isola Rome Italy; ^2^ 1° Clinica Ortopedica, ASST Centro Specialistico Ortopedico Traumatologico Gaetano Pini‐CTO Milan Italy; ^3^ Laboratory of Applied Biomechanics, Department of Biomedical Sciences for Health Università degli Studi di Milano Milan Italy; ^4^ Department of Biomedical, Surgical and Dental Sciences Università degli Studi di Milano Milan Italy; ^5^ Orthopaedic Clinic Lucerne (OKL AG) St. Anna Hospital Lucerne Luzern Switzerland; ^6^ Department of Surgery, Traumatology and Orthopedic Surgery Hospital of Sterzing (SABES‐ASDAA) Vipiteno‐Sterzing Italy; ^7^ Teaching Hospital of the Paracelsus Medizinischen Privatuniversität Salzburg Austria; ^8^ Orthopaedic and Traumatology department University of Medicine, Pharmacy, Sciences and Technology George Emil Palade Targu Mures Romania; ^9^ Orthopaedics and Traumatology Department Ponderas Academic Hospital Bucharest Romania; ^10^ Central Military Emergency Hospital Dr. Carol Davila Bucharest Romania; ^11^ Department of Orthopedics and Traumatology University of Health Sciences, M. S. (Metin Sabancı) Baltalimani Bone and Joint Diseases Research Hospital Istanbul Turkey; ^12^ Département de Chirurgie Orthopédique et de Médecine du Sport, FIFA Medical Center of Excellence Hôpital de la Croix‐Rousse, Centre Hospitalier Universitaire de Lyon Lyon France; ^13^ LIBM – EA 7424, Interuniversity Laboratory of Biology of Mobility Université Claude Bernard Lyon 1 Lyon France; ^14^ U.O.C. Ortopedia e Traumatologia Pediatrica, ASST Gaetano Pini/CTO Milan Italy; ^15^ Cankaya Orthopedics Ankara Turkey; ^16^ Department of Orthopedic Surgery and Traumatology Kantonsspital Baselland Liestal Switzerland; ^17^ Department of Clinical Research, Research Group Michael T. Hirschmann, Regenerative Medicine & Biomechanics University of Basel Basel Switzerland

**Keywords:** extensor mechanism rupture, patient reported outcomes, total knee replacement, treatment options

## Abstract

**Purpose:**

Extensor mechanism (EM) ruptures after total knee arthroplasty (TKA) are rare but lead to substantial functional impairment and morbidity. Treatment remains challenging due to the diversity of surgical techniques and the limited number of comparative studies. The European Knee Associates (EKA) group conducted a systematic review to evaluate available treatments for EM rupture following primary TKA, assess outcomes such as extensor lag, patient‐reported outcome measures (PROMs) and walking status, and to identify prevailing trends and complication rates.

**Methods:**

A systematic review was registered in PROSPERO (CRD42022341591) and conducted according to Preferred Reporting Items for Systematic Review and Meta‐Analysis and Cochrane guidelines. PubMed/Medline and Scopus databases were searched for clinical studies published between 2014 and 2024 reporting outcomes after EM repair in TKA patients. Inclusion criteria encompassed retrospective and prospective studies in English or German. Studies on EM ruptures due to infection, revision TKA, or patellectomy were excluded. Data extraction included demographics, time‐to‐reconstruction, repair type, extensor lag, PROMs and walking status. Due to heterogeneity, a meta‐analysis was not feasible, and results were descriptively reported.

**Results:**

A total of 32 studies comprising 893 EM rupture cases were included. The mean patient age was 66.8 years (standard deviation [SD] ± 7.4), with a mean body mass index of 34.5 (SD ± 6.2). Patellar tendon ruptures (39%) were the most frequent. Nine different surgical techniques were identified, including allografts, synthetic meshes, autografts and direct repairs. The overall complication rate was 16%, with autografts having the highest complication rate (38%). All repair techniques led to improvements in extensor lag and post‐operative Knee Society Scores exceeding the minimal clinically important difference thresholds. However, walking status and PROMs were inconsistently reported.

**Conclusions:**

EM failure after TKA remains a complex complication with variable outcomes depending on treatment strategy. Standardization of surgical techniques and outcome measures, along with multicenter collaborations, is crucial to improve future patient care as the number of TKA procedures continues to rise.

**Level of Evidence:**

Level III, systematic review of Level III studies.

**Abbreviations**
BMI: body mass index
EKA: European Knee Associates
EM: extensor mechanism
KSS: Knee Society Score
LARS: Ligament Augmentation and Reconstruction System
MCID: minimal clinically important difference
MEDLINE: Medical Literature Analysis and Retrieval System Online
PROM: patient‐reported outcome measure
PT: patellar tendon
QT: quadriceps tendon
SCB: substantial clinical benefit
TKA: total knee arthroplasty

## INTRODUCTION

The incidence of extensor mechanism (EM) ruptures in total knee arthroplasty (TKA) is low, ranging between 0.17% and 2.5%. However, this condition can lead to substantial adverse effects for patients [36]. Patella fractures represent nearly 60% of cases, whereas quadriceps tendon (QT) deficiencies constitute 25%. Ruptures of the patellar tendon (PT) are less common, with a prevalence of 12% [18].

The severity and duration of the injury, along with underlying health conditions, influence the morbidity and quality of life impact of EM failure [27].

Upon diagnosis, precise surgical planning necessitates supplementary instrumental exams, such as X‐ray, ultrasound, magnetic resonance imaging and computed tomography. These exams are crucial for accurately pinpointing the lesion's location, assessing the integrity of prosthetic components and adjacent tissues and ruling out underlying infections. Furthermore, these findings assist the surgeon in deciding whether to retain or revise the prosthesis, as a misaligned or infected primary implant can contribute to EM failure [28].

The surgical approach is complex and involves various strategies, all carrying a significant risk of poor functional outcomes and severe complications. Treatment alternatives include direct primary repair using allografts or synthetic ligaments, tissue augmentation with autografts and reconstruction with a complete extensor apparatus allograft [27].

Due to the infrequent occurrence of these lesions and the lack of definitive data, surgeons often employ different strategies for EM reconstruction. Treatment decisions are frequently based on personal experiences or conversations with colleagues, relying on anecdotal evidence. Therefore, there is considerable interest in developing robust, evidence‐based recommendations to enhance the treatment and outcomes for patients experiencing these complications. In response, the European Knee Associates (EKA) section within the European Society for Sports Traumatology, Knee Surgery and Arthroscopy has established a study group dedicated to conducting a systematic literature review. This review aims to evaluate available treatments for acute and chronic EM rupture following primary TKA, assess extensor lag in various rupture scenarios, examine patient‐reported outcome measures (PROMs), evaluate walking status and assess complication rates associated with the different reconstruction techniques.

## METHODS

The study protocol was submitted and officially registered in PROSPERO (CRD42022341591) in July 2022.

Inclusion criteria were all primary clinical studies (including retrospective studies, observational studies and randomized clinical trials) reporting on outcomes following EM rupture repair published in English or German. Exclusion criteria were editorials, biomechanical or cadaveric studies, case reports, EM ruptures secondary to infections, revision TKA, and EM rupture following patellectomy.

Following protocol registration, the systematic literature review was undertaken in December 2024. The authors explored the PubMed/Medline and Scopus databases, using a combination of the following search terms alongside Boolean operators: ‘Knee’, ‘extensor mechanism’, ‘extensor apparatus’, ‘quadriceps’, ‘patella tendon’, ‘patellar tendon’, ‘insufficiency’, ‘deficiency’, ‘rupture’, ‘tear’, ‘total knee arthroplasty’, ‘total knee replacement’, ‘revision total knee arthroplasty’, ‘revision total knee replacement’, ‘repair,’ ‘reconstruction’, and ‘allograft’. The review was bounded by a time frame of 10 years, between 2024 and 2014 and adhered to the Preferred Reporting Items for Systematic Review and Meta‐Analysis and Cochrane guidelines [19, 23, 32].

After the database search, the results were collected in.csv format by two review authors. Duplicates were removed using Microsoft Excel. All included studies underwent title, abstract and full‐text screening by two authors, with any differences resolved through discussion. In instances where consensus could not be achieved, a third reviewer was consulted for a final decision.

**Figure 1:**
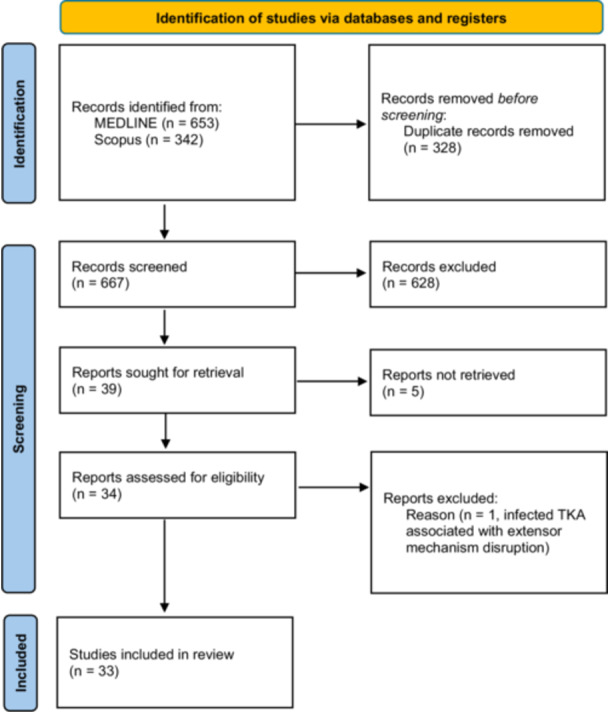
Selection criteria flow chart.

After Title and Abstract screening, 33 prospective and retrospective observational studies analyzing groups of patients treated for extensor apparatus failure in TKA were identified [1, 2, 3, 5, 6, 7, 8, 11, 12, 13, 14, 15, 16, 17, 18, 20, 21, 22, 24, 25, 26, 27, 28, 30, 31, 33, 34, 35, 37, 38, 39, 41, 42]. After full‐text reading, one study was excluded as it focused on marlex‐mesh reconstruction following EM ruptures secondary to TKA infection, leaving 32 studies for data extraction [30] (Figure 1).

Subsequently, two authors independently compiled the relevant data into a Microsoft Excel spreadsheet for further analysis. Any discrepancies in data collection were resolved by a third reviewer. Relevant extracted data points were patient demographics (age, sex, body mass index [BMI] and follow‐up), time‐to‐reconstruction (as time passed from EM rupture until surgical reconstruction), repair type, extensor lag, PROMs and walking status.

No risk of bias assessment was performed, given that all identified studies were observational single‐cohort studies.

### Statistical analysis

Patient demographics were reported using mean and standard deviation (SD) when available. EM ruptures, repair types, and complications were reported as counts and percentages out of the total. Pre‐ and post‐operative extensor lag was reported using the mean reported values when available. Pre‐ and post‐operative Knee Society Score (KSS) values were reported using mean values when available and compared to the already reported minimal clinically important difference (MCID) and substantial clinical benefit (SCB) thresholds. SDs could not be reported, given the increased heterogeneity in reporting. Given the marked detected heterogeneity, effect measures could not be calculated, and therefore, all relevant data points were collected and reported in tabular format.

Microsoft Excel was used to extract and report all relevant data in tabular form.

## RESULTS

A total of 924 cases of EM ruptures were identified. Cases in which the EM failed due to prior patellectomy (*n* = 7), osteosarcoma (*n* = 18) and giant cell tumour (*n* = 6) were intentionally excluded from the analysis, leaving 893 cases for analysis. Demographic data revealed a mean age of 66.8 years (range: 49.7–79.4 years). Gender distribution showed that 65% of the patients (*n* = 371) were female, while 35% (*n* = 203) were male. Mean BMI across all reported studies (*n* = 23) was 34.5 (SD ± 6.2). Mean reported follow‐up was 47 ± 24 months with a minimum of 3 and a maximum of 180 months, respectively.

EM ruptures were recorded to occur at multiple anatomical locations. PT ruptures were the most prevalent, with 39% (357), followed by QT ruptures at 25% (232) and EM ruptures initiated by patellar fractures at 9% (80). However, in 27% (241) of these cases, the actual site of failure was not specified. Consequently, these cases were included in a separate group labelled ‘Supposed full EM ruptures’.

Patients were stratified based on the type of intervention. Nine types of materials were used for reconstructing the ruptured EM (Table 1). The overall complication rate was 16% (96). Pre‐ and post‐operative extensor lag are reported in Tables 2 and 3. The largest pre‐operative extensor lag was present in patients undergoing allograft reconstruction, while patients undergoing end‐to‐end repair by suturing had the smallest pre‐operative extensor lag. Post‐operative extensor lag improved in all cases from pre‐operative values with VY plasty, auto‐graft reconstruction and end‐to‐end repair by suturing having the largest improvements.

**Table 1:** Summary of surgical techniques for EM reconstruction post‐TKA, including indications, case numbers and complication profiles.

Technique	Total cases (*n*)	PT rupture (%)	QT rupture (%)	Patellar fracture (%)	Other/unknown (%)	Complication rate (%)	Complications
Marlex mesh	148	38% (56)	24% (36)	7% (10)	31% (46)	19% (28)	8 infections (5%), 5 amputations (3%), 5 symptomatic lengthening (3%), 5 QT re‐ruptures (3%), 5 PT re‐ruptures (3%), 1 arthrodesis (1%)
Allograft	286	35% (101)	6% (18)	10% (28)	48% (137)	14% (41)	14 symptomatic lengthening (5%), 13 infections (4%), 3 arthrodesis (1%), 3 QT re‐ruptures (1%), 4 PT re‐ruptures (1%), 1 each of CRPS, patellar fracture, poor flexion, and failed fixation (<1%)
End‐to‐end repair	68	28% (19)	54% (37)	18% (12)	‐	35% (24)	20 infections (29%), 3 arthrodesis (4%), 1 amputation (1%)
VY plasty	46	‐	100% (46)	‐	‐	7% (3)	1 each: QT re‐rupture (2%), symptomatic lengthening (2%), arthrodesis (2%)
Autograft	32	50% (16)	50% (16)	‐	‐	38% (6)	6 symptomatic lengthening (38%)
LARS	10	40% (4)	50% (5)	10% (1)	‐	‐	‐

Abbreviations: CRPS, complex regional pain syndrome; EM, extensor mechanism; LARS, Ligament Augmentation and Reconstruction System; PT, patellar tendon; QT, quadriceps tendon; TKA, total knee arthroplasty.

**Table 2:** Pre‐ and post‐operative mean values of extensor lags reported for end‐to‐end repair by suturing, VY‐plasty, autograft and LARS reconstruction.

Type of repair	Preoperative extensor lag (°)	Post‐operative extensor lag (°)
End‐to‐end repair (suture) (*n* = 121)^a^	11	5.6
VY‐plasty (*n* = 58)^b^	42.8	2.3
Autograft (*n* = 25)^c^	42.5	5.3
LARS (*n* = 10)^d^	0	18

Abbreviation: LARS, Ligament Augmentation and Reconstruction System.

^a^ Three studies repaired extensor mechanism (EM) ruptures by end‐to‐end suturing [11, 12, 22], but only two reported the pre‐ and post‐operative extensor lag [12, 26].

^b^ Three studies reported results of VY‐plasty for EM ruptures [6, 38], but only one study reported pre‐ and post‐operative extensor lag [6, 38].

^c^ There were three studies reporting autograft reconstruction of EM ruptures [21, 39]. Two studies reported the mean preoperative and post‐operative extensor lag values [21, 37] while the other reported median values [39].

^d^ In LARS reconstruction, no preoperative extensor lag was reported, but one study reported a mean post‐operative extensor lag of 18° [31].

**Table 3:** Pre‐ and post‐operative extensor lags for Achilles tendon bone block allograft (ATA), full extensor mechanism (EM) allograft and synthetic mesh reconstructions were described.

Type of repair^a^		Preoperative mean extensor lag (°)	Post‐operative mean extensor lag (°)
ATA (*n* = 89)	Mean	55.86	12.50
	Range	35−90	2−26
Full EM allograft (*n* = 244)	Mean	56.85	16.47
	Range	32−90	1−45
Synthetic mesh (*n* = 289)	Mean	34.18	12.38
	Range	21–47.9	3–26

^a^ Studies were further divided into three categories, reporting mean pre‐ and post‐operative extensor lag results following ATA reconstruction, full EM allograft and synthetic mesh reconstruction.

Pre‐ and post‐operative KSS are reported in Table 4. All repair types achieved post‐operative KSS values above the MCID threshold. Post‐operative SCB was obtained, depending on the study, in end‐to‐end repair by suturing, gastrocnemius flap, marlex mesh, Achilles tendon bone block allograft, and full EM allograft reconstructions.

**Table 4:** Pre‐ and post‐operative reported mean Knee Society Scores (KSS) divided by type of repair.

Reported outcome scores	Values		Author
	Pre	Post	
End‐to‐end repair by suturing			
KSS	85	125.7	[12]
VY plasty			
KSS‐knee/KSS‐function	57.6/52.8	81.3/78/7	[25]
KSS‐knee/KSS‐function	44.5/45.6	88.7/87.5	[38]
Gastrocnemius flap			
KSS	38	117	[20]
Marlex mesh			
KSS	32	72	[1]
KSS‐knee/KSS‐function	41.5/14.6	79.5/64.2	[5]
Autograft			
KSS	40	65	[21]
Achilles tendon bone block allograft			
KSS	32	128	[4]
KSS‐knee/KSS‐function	46/27	133/76	[10]
KSS	34.9	87.7	[21]
KSS	40	67	[24]
Full EM allograft			
KSS	32	128	[4]
KSS	49	39.4	[10]
KSS	52	88	[10]
KSS	46	133	[9]
KSS‐knee/KSS‐function (*n* = *X*) refs	27/19	76/57	[12]
KSS‐knee/KSS‐function	52.6/36.6	74.3/59.4	[14]
KSS	45	138	[15]
KSS	34	78.9	[21]
KSS	40	67	[24]
KSS	101	116	[34]

Patient's walking status after EM repair is reported in Table 5.

**Table 5:** Walking status as reported in each study.

Walking status	
Author	Description
End‐to‐end repair by suturing	
	*Not reported*
VY plasty	
[25]	All patients (*n* = 21) except one, who had a post‐operative infection, were pain‐free and able to walk without assistive devices at the final follow‐up.
[38]	Nineteen (83%) of the 23 patients reached pre‐rupture levels of activity, and all patients were able to walk without assistive devices at the time of the latest follow‐up.
[6]	Not reported. The authors did note that one patient, who had a chronic quadriceps tendon rupture without a prior TKA, showed significant improvement in walking ability after the surgery. This patient transitioned from being dependent on a walker to using a cane for mobility.
Marlex mesh	
[1]	Not reported
[5]	All patients (*n* = 12) were walking, with nine requiring occasional use of a cane, one needing two canes full‐time, one with a hinged brace, and one without any assistive devices. Two patients were unable to ascend and descend stairs, leading to a low post‐operative KS function score.
[8]	At the time of final follow‐up, among successful reconstructions (*n* = 19), five patients were ambulating independently, five were ambulating with a cane, five were ambulating with a walker, and four were able to participate in transfers but were predominantly using a wheelchair.
[16]	Not reported
Autograft	
[39]	All patients (*n* = 9) had returned to ordinary daily and working activities. None of the patients perceived the loss of knee flexion at the latest follow‐up as interfering with their activities of daily living. Five patients walked without aids, four with a cane, and no patients walked with crutches. No patients used a brace, and they were all able to ascend and descend stairs without problem.
[37]	All patients regained community ambulation. However, around one third in both groups (33% in the peroneal graft group, 28% in the mesh group) required walking aids like canes or walkers. Walking ability was comparable between groups.
Achilles tendon bone block allograft	
[13]	Seventeen cases presented satisfactory results (58.6%). Eleven cases were considered reconstruction failures (37.9%), and one case was lost to follow‐up.
[24]	50% of the patients had their walking status improve after surgery, and 50% had a stationary walking status. In no patient did the walking status decrease to a lower level of activity.
[41]	Post‐operatively, all patients (n = 16) required an assistive device for ambulation.
Full extensor mechanism (EM) allograft	
[24]	50% of the patients (*n* = 8) had their walking status improve after surgery, and 50% had a stationary walking status (*n* = 8). In no patient did the walking status decrease to a lower level of activity.
[2]	Walking ability was significantly better in patients with successful EM reconstruction. While 69.1% of all patients needed walking aids at final follow‐up, only 53.6% of those with a successful outcome required them, compared to 85.2% in the failure group (*p* < 0.01). This highlights the impact of successful reconstruction on improving post‐operative mobility.
[3]	Not reporting on ambulatory aid use specifically, the modest improvement in extensor lag and lack of significant change in PROMs suggest that mobility and independence were not fully restored. Overall, the study highlights the limited benefit and durability of EMR, especially in complex or revision cases.
[35]	Not reported
[18]	No significant difference in ambulatory status between the two groups (*p* = 0.34) at the most recent follow‐up. However, the study does not provide detailed information on the specific walking abilities or the use of assistive devices among patients.
Proximal tibia‐patellar tendon composite allograft	
[33]	Post‐operatively, all patients (24 included patients and 2 excluded because of limb amputation, leaving 22 patients for this discussion) were community ambulators. Four patients walked without the use of supports, 10 patients used a cane for safety while walking, and 8 patients required the continuous use of one cane or crutch for walking. One patient had no discernible limp, 17 patients had a minor cosmetic limp, and 2 patients had a major cosmetic limp. Among the 14 working patients, 5 were able to return to their former occupations. Nine other patients were students, and one was retired.

Abbreviations: EMR, endoscopic mucosal resection; PROM, patient‐reported outcome measure.

## DISCUSSION

The primary finding of this study is the multitude of surgical options available for treating extensor apparatus failure in TKA. The variability in presentation and the limited number of cases in clinical practice present challenges in creating a large series of treated patients. The distinction between acute and chronic EM deficiencies lacks a clear definition, with authors presenting different timing and clinical manifestations. However, surgical preference is evidently influenced by the time elapsed since the injury. The results of this study suggest a preference for allografts in cases with a longer time since the injury. Synthetic meshes, on the other hand, are utilized even later in disease progression, at an average of 10.5 months post‐EM disruption. Establishing a more standardized protocol for graft selection based on the time from failure could prove valuable for knee surgeons in the future. At present, there are no clear indications guiding the choice between allografts and synthetic meshes. It seems to depend solely on the expertise of each surgical centre. This aspect also raises clinical and healthcare economic considerations, given the substantial cost difference between allografts and synthetic meshes. In a cost‐effectiveness study comparing synthetic and allograft used for EM repair after total knee replacement, the allograft cost was significantly higher compared to the synthetic graft cost (*p* = 0.001). The mean total cost was 4733.08 Canadian dollars for the synthetic group and 24,050.40 Canadian dollars for the allograft group (*p* = 0.17) [42]. Newer studies [18, 35] suggest similar outcomes between synthetic mesh and allograft reconstructions, potentially favouring mesh in cost‐sensitive settings.

However, in the studies included in this review, there appears to be a clear cut‐off between time to reconstruction and type of repair. On average, up to 2 months after EM disruption, surgeons tend to employ repairs with or without autograft augmentation, while after 2 months, allografts and synthetic meshes are the treatment of choice. However, not all studies report time‐to‐reconstruction, so it is difficult to draw clear conclusions on what should be considered acute or chronic EM disruption.

It is noteworthy that there is currently no reliable method for predicting the likelihood of EM failure after total knee replacement, as reported by some authors. However, there have been advancements in implant alignment, rotation and soft‐tissue balance, as well as improvements in the design of femoral implants and patellar components, which have resulted in a reduction in EM complications. Identifying and addressing risk factors, as well as implementing preventive measures, could potentially reduce the incidence of EM failure and minimize the need for complex procedures and their potential negative impact on patient outcomes [29].

It is important to note that patient comorbidities can significantly impact the outcomes of surgical treatments. Nonetheless, it should be mentioned that a study by Courtney et al. [12] found that a previous history of prosthetic joint infection and a chronic (>2 weeks) injury were associated with a higher risk of poor outcomes following allograft EM reconstruction, as determined by multivariate analysis [10]. All of these aspects demand careful consideration within the knee surgeons' community, particularly in light of the anticipated significant increase in the number of knee arthroplasty procedures in the coming years, with a concomitant rise in the incidence of complications. The increasing prevalence of knee osteoarthritis has been the subject of an informative cohort study comparing the prevalence of knee OA in early industrial and post‐industrial populations, which found that knee OA was present in 16% of the post‐industrial sample, but only 6% of the early industrial population. After controlling for age, BMI and other variables, knee OA prevalence was found to be 2.1‐fold higher (95% confidence interval: 1.5–3.1) in the postindustrial sample than in the early industrial sample [40]. Demographic data also reveal that while these lesions are expected to be present in older patients, the age range indicates that some patients may be as young as 50 years old.

When considering surgical techniques, it is difficult to determine the type of EM lesions that occurred after primary TKA or revision TKA in the studies included in this review, as most studies report mixed series of patients. Therefore, only qualitative considerations can be proposed by the authors. Depending on the site of EM failure, trends in surgical preferences can be observed. For instance, PT disruptions, which are known to be generated after primary or revision TKA and are difficult to heal, tend to be addressed by allograft repair. On the other hand, QT disruptions are typically addressed by primary repair, which suggests that a quadriceps snip is expected to heal very well, while a PT avulsion, even if it occurs during primary TKA, is expected to have worse outcomes, with a higher risk of re‐rupture and re‐operation. Patellar fractures are also a common cause of EM failure. In the acute setting, primary repair is performed, but if the repair fails, we find ourselves in a grey area where synthetic meshes or allografts may be used to address these lesions, depending on each centre's expertise.

Regarding the population affected by full EM failure in total knee replacement, this review found a significant difference in the prevalence of extensor apparatus disruption between males and females, with a significant prevalence in the female population, and the mean BMI across all studies of 34.5, suggesting that patients suffering from obesity are most frequently affected.

Preoperative and post‐operative extensor lags are not reported in all studies, so we have only included cases where these values are reported, clustered by the type of repair rather than the type of lesion, as many lesions fall under the same treatment option. An interesting conclusion for extension lag was drawn by Burnett et al., who investigated two tensioning techniques for full EM reconstruction [10]. In one cohort, the EM was lightly tensioned to allow 60° of passive intraoperative flexion, while in the second cohort, the EM was tightly tensioned in full extension to allow no more than 20–30° of passive intraoperative flexion. Their results indicate that when utilizing EM allograft for EM reconstruction, one should anticipate post‐operative loosening of the graft. Therefore, the graft can be tightly tensioned in full extension. Reported outcome scores also vary across studies, but the most commonly used score is KSS. The main issue we encountered while extracting a mean KSS score corresponding to each treatment type was that some studies reported the overall KSS score, while others reported the knee and function subscales. Walking status, an important measure of surgical success following EM failure, was inconsistently reported. Nevertheless, we included reports on what each author considered as ‘walking status’ to emphasize that currently, there is no standardized method to assess post‐operative outcomes in the context of everyday living for patients suffering from ‘EM failures’.

This study has some limitations. First of all, the included studies had different inclusion and exclusion criteria in many cases, without valid randomization of cases and comparable outcomes. This made data analysis difficult, and for all outcomes, a meta‐analysis was not possible. Another limitation is the heterogeneity of the site and time of failure in the patients included in the studies, making it difficult to compare surgical procedures and outcomes. A third limitation is the inconsistent use of the KSS. A mean value was difficult to estimate because some studies report results on each subsection of the original score (knee and function subsections), while others report the composite KSS value. Therefore, we preferred to present the mean reported value when available. Furthermore, SDs were inconsistently reported, making the estimation of a mean SD across each repair type impractical. A fourth limitation is that walking status has no standardized reporting measure; therefore, each study reported what was considered important at that time and therefore, summarizing walking status for each repair type was not feasible.

Despite its limitations, the study's strength lies in consolidating various reconstructions available for EM rupture and their expected mean outcomes into a single reference. Moreover, it underscores the importance of standardizing outcome measures for this patient group to facilitate meaningful comparisons among surgical experts dealing with this challenging pathology.

## CONCLUSION

EM failure following TKA remains a rare but severe complication with considerable functional consequences. This systematic review highlights the wide variability in surgical strategies, outcome reporting, and complication profiles associated with EM reconstruction. Importantly, the data suggest a practical distinction between acute and chronic ruptures, with a temporal threshold of approximately two months guiding treatment choice: direct repair or autograft reconstruction is more commonly used in acute settings, whereas allografts and synthetic meshes are favoured for chronic or structurally deficient cases. Direct repairs were associated with higher complication rates, particularly infections and surgical failures, indicating that they should be limited to carefully selected patients with good tissue quality.

While the heterogeneity of current literature precludes meta‐analysis, this review underscores the urgent need for standardized definitions (e.g., timing of rupture, outcome measures such as extensor lag, PROMs and walking status) and treatment algorithms. Beyond calling for future guidelines, our findings support the development of preliminary clinical protocols tailored to rupture timing, anatomical location and patient‐specific factors to inform surgical decision‐making and improve outcomes in this challenging patient population.

## AUTHOR CONTRIBUTIONS

**Bruno Violante**: Conceptualization; project administration; supervision. **Artur Kroell**: Investigation; methodology; resources; validation; writing—original draft; writing—review and editing. **Riccardo Compagnoni**: Investigation; methodology; resources; validation; writing—original draft, writing—review and editing. **Michael Engl**: Investigation; methodology; resources; validation; writing—original draft; writing—review and editing. **Octav Russu**: Data curation; formal analysis; investigation; visualization; writing—original draft. **George M. Avram**: Data curation; formal analysis; investigation; visualization; writing—original draft. **Sarper Gursu**: Data curation; formal analysis; investigation; visualization; writing—original draft. **Elvire Servien**: Writing—review and editing. **Pietro Simone Randelli**: Investigation; methodology; resources; validation; writing—review and editing. **Reha Tandogan**: Conceptualization; project administration; supervision. **Francesco Puglia**: Data curation; formal analysis; investigation; visualization; writing—original draft. **Michael T. Hirschmann**: Conceptualization; project administration; supervision.

## CONFLICT OF INTEREST STATEMENT

The authors declare no conflicts of interest.

## ETHICS STATEMENT

No ethical approval was required to conduct the systematic literature search and data processing. The systematic review was conducted according to a preregistered protocol published in PROSPERO (CRD42022341591).

## Data Availability

This is a literature review. Data included in this article comes from a published manuscript available on PubMed/Medline.
